# Molecular Mechanisms for Biliary Phospholipid and Drug Efflux Mediated by ABCB4 and Bile Salts

**DOI:** 10.1155/2014/954781

**Published:** 2014-07-15

**Authors:** Shin-ya Morita, Tomohiro Terada

**Affiliations:** Department of Pharmacy, Shiga University of Medical Science Hospital, Otsu, Shiga 520-2192, Japan

## Abstract

On the canalicular membranes of hepatocytes, several ABC transporters are responsible for the secretion of bile lipids. Among them, ABCB4, also called MDR3, is essential for the secretion of phospholipids from hepatocytes into bile. The biliary phospholipids are associated with bile salts and cholesterol in mixed micelles, thereby reducing the detergent activity and cytotoxicity of bile salts and preventing cholesterol crystallization. Mutations in the *ABCB4* gene result in progressive familial intrahepatic cholestasis type 3, intrahepatic cholestasis of pregnancy, low-phospholipid-associated cholelithiasis, primary biliary cirrhosis, and cholangiocarcinoma. *In vivo* and cell culture studies have demonstrated that the secretion of biliary phospholipids depends on both ABCB4 expression and bile salts. In the presence of bile salts, ABCB4 located in nonraft membranes mediates the efflux of phospholipids, preferentially phosphatidylcholine. Despite high homology with ABCB1, ABCB4 expression cannot confer multidrug resistance. This review summarizes our current understanding of ABCB4 functions and physiological relevance, and discusses the molecular mechanism for the ABCB4-mediated efflux of phospholipids.

## 1. Introduction

**Figure 1:**
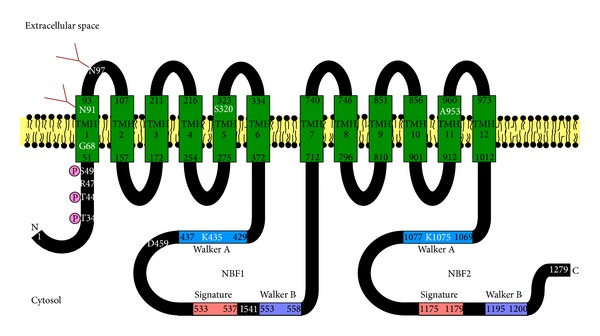
Schematic model of ABCB4. ABCB4 consists of twelve TMHs spanning the plasma membrane and two cytosolic NBFs containing the Walker A, Walker B, and signature motifs. N and C indicate the N- and C-termini of ABCB4, respectively. TMHs are predicted from the crystal structure of mouse Abcb1a (PDB code 3G61) reported by Aller et al. [60]. The residues N91 and N97 are *N*-glycosylated with complex oligosaccharides. The residues T34, T44, and S49 are phosphorylation sites. The T34M, R47G, K435M, and K1075M mutations impair the phospholipid efflux activity of ABCB4. The G68H, S320F, D459H, I541F, and A953D mutations result in the intracellular retention of ABCB4.

**Figure 2:**
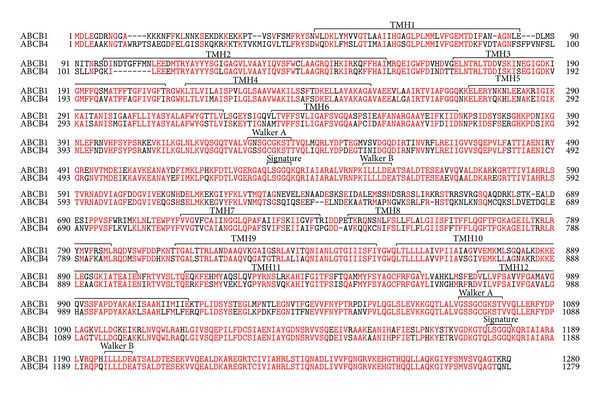
Amino acid sequence alignment of human ABCB1 and ABCB4. Identical amino acids are* red*. Functional domains are shown above the sequence alignment. TMHs are predicted from the crystal structure of mouse Abcb1a (PDB code 3G61) reported by Aller et al. [60].

ABCB4, also called multidrug resistance 3 (MDR3), is a 1279-amino acid transmembrane protein. ABCB4, belonging to the ATP-binding cassette (ABC) transporter family, consists of two homologous halves, each of which contains six transmembrane helices (TMHs) and a cytoplasmic nucleotide-binding fold (NBF) (Figure 1) [1]. ABCB4 has two *N*-glycosylation consensus sites in the first extracellular loop. The human* ABCB4* gene on chromosome 7q21.1 has 28 exons and 27 introns and is located adjacent to the* ABCB1* gene [2]. ABCB1, also called MDR1 or P-glycoprotein, has a 1280-amino acid sequence with 76% identity and 86% similarity to ABCB4 (Figure 2). ABCB1 is normally present in various tissues, including the liver, kidney, intestinal mucosa, and capillary endothelial cells at the blood-brain barrier [3]. On the other hand, ABCB4 protein is mainly expressed in the liver, although low levels of ABCB4 mRNA expression are found in the adrenal gland, muscle, tonsil, spleen, placenta, testis, and ileum [4]. ABCB1 exports a large number of structurally unrelated hydrophobic compounds and is responsible for multidrug resistance of cancer cells. However, ABCB4 is unable to export most ABCB1 substrates efficiently and to confer equivalent multidrug resistance properties [5]. In the liver, ABCB4 is localized to the canalicular membranes of hepatocytes and is necessary for the secretion of phospholipids into bile.

## 2. Discovery of ABCB4

In 1976, Juliano and Ling demonstrated the overproduction of a large membrane protein called P-glycoprotein in multidrug resistant cells [6]. In 1986, Roninson et al. found two kinds of MDR gene, called* MDR1* and* MDR2*, amplified in multidrug resistant cell lines from KB cells [7]. Ueda et al. have shown that a full-length cDNA for the* MDR1* gene encodes P-glycoprotein and confers multidrug resistance phenotype [8, 9]. In 1987, van der Bliek et al. isolated the human* MDR3* gene from liver cDNA libraries and determined its sequence [1, 10].* MDR2* is actually identical in sequence to* MDR3* [1].

## 3. Physiological and Pathophysiological Roles of ABCB4

The function of biliary phospholipid secretion is to protect the membranes of cells facing the biliary tree against bile salts. Biliary phospholipids also play a key role in solubilizing cholesterol. The complexation of bile salts with phospholipids and cholesterol into mixed micelles strongly reduces the cytotoxic detergent effect of bile salts. The concentration of bile salts as monomers and simple micelles is responsible for the potentially damaging effects on membrane bilayers [11]. The hepatocyte plasma membrane is functionally divided into an apical region adjacent to the bile canalicular lumen and a basolateral region in contact with sinusoidal blood. The major structural phospholipids in the outer leaflet of canalicular membranes are phosphatidylcholine (PC) and sphingomyelin (SM) [12]. In bile, however, the predominant (~95%) phospholipid is PC, while SM is present only in trace amounts [13].

Mouse Abcb4, formerly known as Mdr2, is the homolog of human ABCB4. In 1993, Smit et al. generated the mice with homozygous disruption of the* Abcb4* gene, which suffer from liver disease characterized by severe necrotic damage of hepatocytes, strong portal inflammation, and proliferation and destruction of the canalicular and small bile ductular tracts [14]. Spontaneous gallstone formation is also a feature of the phenotype of* Abcb4* knockout mice [15]. These* Abcb4* knockout mice show almost complete absence of PC from their bile, although their bile salt secretion is normal in these mice, suggesting that Abcb4 is required for the secretion of phospholipids into bile [14]. In addition, the cholesterol secretion is strongly suppressed in these mice [14].

Elferink et al. have investigated the relationships among the biliary secretion of bile salt, phospholipids, and cholesterol using* Abcb4* knockout mice [16, 17]. In wild-type mice (+/+), the biliary phospholipid secretion increases with increasing bile salt secretion, and a curvilinear relationship between phospholipid and bile salt secretion is observed [16]. In* Abcb4* homozygous (−/−) mice, the phospholipid secretion is negligible at all bile salt output rates [16]. The bile in wild-type (+/+) mice contains almost exclusively PC with a small amount of phosphatidylethanolamine (PE), but no SM can be detected [17]. The cholesterol secretion does not differ between wild-type (+/+) and* Abcb4* heterozygous (+/−) mice, although the phospholipid secretion rate in* Abcb4* (+/−) mice is 30–50% lower than that in wild-type (+/+) mice [16]. In* Abcb4* (−/−) mice, the biliary secretion of cholesterol was very low [16]. However, the cholesterol secretion in* Abcb4* (−/−) mice is completely restored by infusion of a sufficiently hydrophobic bile salt, taurodeoxycholate, to allow solubilization of cholesterol in the absence of phospholipids [17]. Mixed micelles of bile salts and phospholipids have a much higher capacity to take up cholesterol than simple bile salt micelles [18], and the cholesterol secretion in the absence of phospholipids depends on the cholesterol-solubilizing capacity of the secreted bile salts [17]. These results provide the first evidence that the biliary cholesterol secretion is at least partially independent of ABCB4.

Human ABCB4 mutations result in a wide spectrum of phenotypes, ranging from progressive familial intrahepatic cholestasis type 3 (PFIC3) to adult cholestatic liver disorders [19]. PFIC3 is characterized by high *γ*-glutamyl transpeptidase and early onset of persistent cholestasis that progresses to cirrhosis and liver failure before adulthood [20, 21]. In many cases of PFIC3, liver transplantation is the only therapy. The biliary phospholipid level in PFIC3 patient is dramatically decreased despite the presence of bile acids [20]. This cholestasis may be caused by the toxicity of detergent bile salts that are not associated with phospholipids, leading to bile canaliculus and biliary epithelium injuries. ABCB4 defect is also involved in intrahepatic cholestasis of pregnancy (ICP), low-phospholipid-associated cholelithiasis (LPAC), and primary biliary cirrhosis [4, 22]. ICP is a reversible form of cholestasis in the third trimester of pregnancy and rapidly ameliorated after childbearing. LPAC is characterized by intrahepatic hyperechoic foci, intrahepatic sludge, or microlithiasis [23]. The absence of biliary phospholipids may lead to the destabilization of micelles and promote the lithogenicity of bile with the crystallization of cholesterol. The association between cholangiocarcinoma, a rare malignant tumor of the biliary tract, and ABCB4 mutations has been recently reported [24]. Chronic biliary inflammation may increase cholangiocyte turnover, leading to the growth of altered cholangiocytes and increased susceptibility to cholangiocarcinoma.

**Figure 3:**
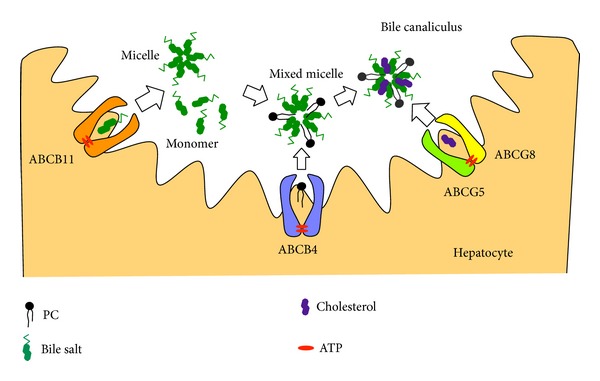
Formation of mixed micelles of bile salts, phospholipids, and cholesterol in bile canaliculus. ABCB11 mediates the efflux of bile salts into bile. The bile salt monomers are essential for the phospholipid efflux mediated by ABCB4. In the presence of mixed bile salt/phospholipid micelles, ABCG5/ABCG8 heterodimer mediates the efflux of cholesterol.

Besides ABCB4, several ABC transporters expressed on the canalicular membranes of hepatocytes are involved in the secretion of lipids and thus in canalicular bile formation (Figure 3). ABCB11, also called bile salt export pump, is implicated in most of the bile salt transport from hepatocytes into the bile canalicular lumen [25]. PFIC2 is caused by mutations in ABCB11 [25]. Patients with PFIC2 usually suffer from severe cholestasis and severe pruritus, with markedly elevated serum bile acids and normal serum *γ*-glutamyl-transferase activity.

ABCG5 and ABCG8 are responsible for the secretion of biliary cholesterol [26, 27]. ABCG5 and ABCG8 form a heterodimer in the endoplasmic reticulum, which is required for their movement into the Golgi and onto the apical membranes [28]. Disruption of the* Abcg5* and* Abcg8* genes in mice strongly decreases the biliary cholesterol secretion but results in modest nonsignificant reductions in the biliary phospholipid levels [27]. On the other hand, Abcg5 and Abcg8 independent routes at least partially contribute to the biliary secretion of cholesterol [29]. The expression of the human* ABCG5* and* ABCG8* transgenes does not increase biliary cholesterol in* Abcb4* knockout mice, suggesting that ABCG5 and ABCG8 require ABCB4 for the secretion of cholesterol into bile [30]. The mixed bile salt/phospholipid micelles generated by ABCB11 and ABCB4 are probably essential for the cholesterol secretion mediated by ABCG5/ABCG8.

Abcb4 protein is also expressed in mouse macrophages. Bone marrow transplantation replaces all bone marrow-derived cells. Pennings et al. have created mice specifically lacking Abcb4 in bone marrow-derived cells, including macrophages, by bone marrow transplantation in* LDLr* knockout mice [31]. Abcb4 deficiency in bone marrow-derived cells leads not only to lower serum cholesterol levels but also to an increase in the atherosclerotic lesion size, suggesting an important atheroprotective function of bone marrow-derived Abcb4 [31]. However, there is no difference in the cholesterol or phospholipid efflux to HDL or apolipoprotein A-I between* Abcb4* (+/+) and* Abcb4* (−/−) macrophages [31].

## 4. ABCB4-Mediated Efflux of Fluorescence-Labeled Phospholipids

**Figure 4:**
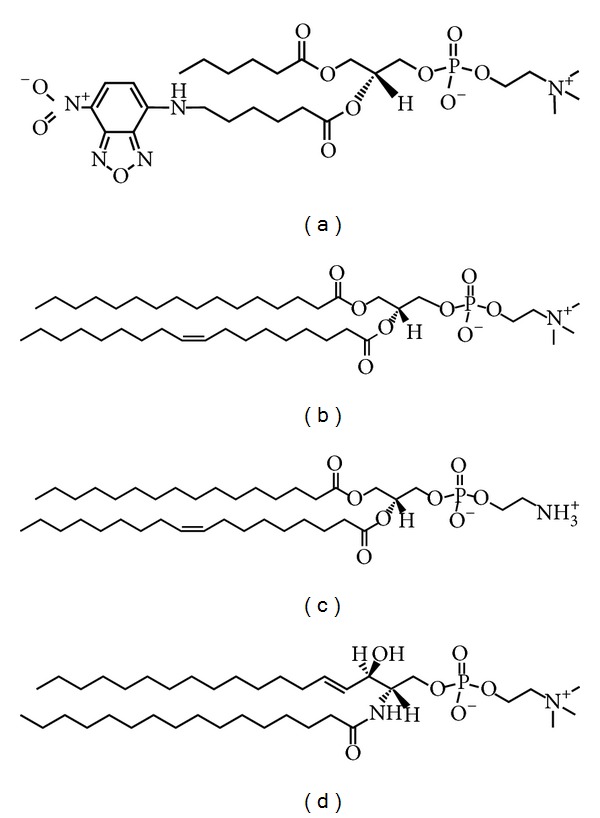
Phospholipid substrates for ABCB4. (a) C_6_-NBD-PC, (b) PC, (c) PE, and (d) SM.

Ruetz and Gros have shown that the expression of mouse Abcb4 in secretory vesicles from the yeast mutant* sec6-4* enhances the translocation of a fluorescence-labeled short-chain PC analog, C_6_-NBD-PC (Figure 4), from the outer to the inner leaflet of the vesicle bilayer, suggesting the function of Abcb4 as a phospholipid translocase [32]. Dithionite, a membrane-impermeant anion, has been used to reduce only C_6_-NBD-PC molecules on the outer leaflet, but not inner leaflet, of the bilayer to their nonfluorescent derivatives. Increased Abcb4-mediated translocation of C_6_-NBD-PC is strictly dependent on ATP and Mg^2+^ and abrogated by the ATPase inhibitor, vanadate, and the ABCB1 modulator, verapamil [32]. Addition of the bile salt taurocholate results in an enhancement of Abcb4-mediated PC translocation activity in the secretory vesicles, suggesting that the stimulation of Abcb4 activity is provoked by the formation of intravesicular aggregates or mixed micelles of taurocholate and C_6_-NBD-PC [33].

**Figure 5:**
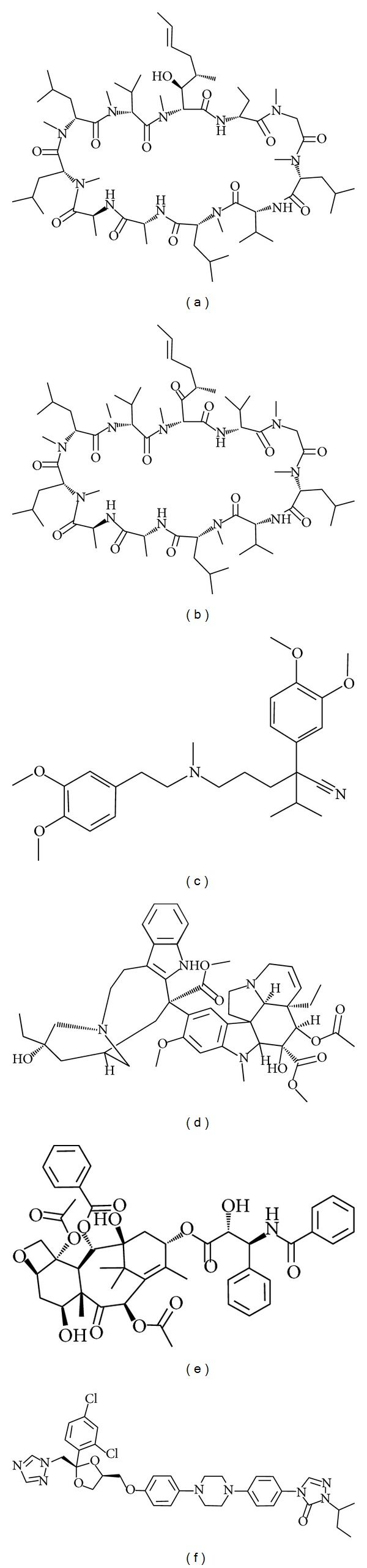
Inhibitors of ABCB4: (a) cyclosporine A, (b) valspodar, (c) verapamil, (d) vinblastine, (e) paclitaxel, and (f) itraconazole.

Van Helvoort et al. have made the stable transfectants of pig kidney epithelial LLC-PK1 cells expressing human ABCB4, which is localized in the apical membranes [34]. C_6_-NBD-diacylglycerol is efficiently converted by cells to the homologous C_6_-NBD-PC and C_6_-NBD-PE, while C_6_-NBD-ceramide yields the corresponding SM (C_6_-NBD-SM). The newly synthesized C_6_-NBD-PC, but not C_6_-NBD-PE or C_6_-NBD-SM, is exclusively secreted to the apical albumin-containing medium of ABCB4-expressing LLC-PK1 cells [34]. The energy depletion markedly reduces the apical release of C_6_-NBD-PC mediated by ABCB4 [34]. Cyclosporine A, valspodar, verapamil, vinblastine, and paclitaxel (Figure 5), but not digoxin, decrease the rate of C_6_-NBD-PC secretion by ABCB4-expressing LLC-PK1 cells [35]. On the other hand, ABCB1-expressing cells secrete C_6_-NBD-PC and C_6_-NBD-PE, but not C_6_-NBD-SM, to the apical albumin-containing medium [34]. Nevertheless, ABCB1 has quite low, if any, ability to mediate the secretion of endogenous long-chain phospholipids into bile. Indeed,* Abcb4* knockout mice do not secrete any phospholipids into bile, despite the substantial expression of* Abcb1a* and* Abcb1b* on the canalicular membranes of hepatocytes.

ABCB4 is expressed in well-differentiated human hepatoblastoma HepG2 cells and distributed to the pseudocanaliculi formed between adjacent cells. The functional activity of ABCB4 has been estimated by the transport of C_6_-NBD-PC into the pseudocanaliculi of HepG2 cells [36]. Additionally, in collagen sandwich cultured rat hepatocytes, the fluorescence-labeled PC is secreted into the bile canaliculi [37].

## 5. ABCB4-Mediated Efflux of Endogenous Phospholipids

The above-mentioned studies have assessed the function of ABCB4 by using C_6_-NBD-PC as a substrate. However, C_6_-NBD-PC is structurally different from the endogenous phospholipids in cells, and its bulky fluorescent probe probably affects the recognition by the transporters. Actually, ABCB1 is not involved in the biliary phospholipid secretion but can transport C_6_-NBD-PC molecules. Hence, to further clarify the function of ABCB4, we have established HEK293 cells stably expressing ABCB1 or ABCB4 and have quantified the efflux of endogenous phospholipids from cells by using enzymatic assays [38]. There is no significant difference in the phospholipid efflux into the medium among the host HEK293 cells, ABCB1-expressing cells, and ABCB4-expressing cells [38]. However, the phospholipid efflux from ABCB4-expressing cells is remarkably enhanced by the addition of taurocholate, while the phospholipid efflux from ABCB1-expressing cells is not affected by taurocholate [38]. The phospholipid efflux mediated by ABCB4 is increased with increasing concentrations of taurocholate and shows concentration dependence from 0.2 mM to 1 mM taurocholate [38]. The critical micelle concentration of taurocholate is estimated to be 2.5 mM in the medium by light scattering measurements, and thus, below 2.5 mM, taurocholate molecules are present as monomers [38]. Based on these results, the monomer forms of bile salts most likely function, at least initially, in supporting the ABCB4-mediated phospholipid efflux. The majority of bile salts are conjugated in the liver to glycine or taurine, which increases their polar surface. The phospholipid efflux from ABCB4-expressing cells increases in the order of taurocholate > glycocholate > cholate [38], which is inversely correlated with the bile salt hydrophobicity index, from the least to the most hydrophobic: taurocholate (0) < glycocholate (+0.07) < cholate (+0.13) [39]. ABCB4-K435M and ABCB4-K1075M, Walker A lysine mutants in NBFs, do not mediate the phospholipid efflux in the presence of taurocholate, suggesting that ATP hydrolysis is essential for the ABCB4-mediated efflux [38]. Verapamil also completely blocks the taurocholate-dependent efflux of phospholipids from ABCB4-expressing cells, suggesting that ABCB1 and ABCB4 have quite similar substrate binding domains [38]. Mass spectrometry has revealed that ABCB4-expressing cells preferentially secrete PC (16:0-16:1 PC, 16:0–18:2 PC, 16:0–18:1 PC, and 18:0–18:2 PC) rather than SM (16:0–18:1 SM) in the presence of taurocholate [38]. Moreover, we have developed enzyme-based fluorometric methods for quantifying PC, PE, and SM, which are simple, rapid, and sensitive and have high throughput [40, 41], and have demonstrated that the addition of taurocholate significantly increases the efflux of PC, PE, and SM from ABCB4-expressing cells, although the enhancement of PE or SM efflux by taurocholate was less marked than that of PC efflux [42]. Notably, apolipoprotein A-I or HDL cannot stimulate the ABCB4-mediated phospholipid efflux, despite their ability to accept phospholipids from ABCA1-expressing cells [31, 43], whereas taurocholate also promotes the phospholipid efflux mediated by ABCA1 [43].

Gautherot et al. have recently identified two point mutations of the ABCB4 N-terminal domain, T34M and R47G, in patients with LPAC or ICP [44]. The PC secretion activities of both mutants in HEK293 cells are low compared with that of wild-type ABCB4 [44]. In the N-terminal domain of ABCB4, Thr34, Thr44, and Ser49 are phosphorylatable [44]. The T34M mutation directly abolishes the phosphorylation, while the R47G mutation indirectly impairs the phosphorylation of Thr44 or Ser49 [44]. The ABCB4-mediated secretion of PC is enhanced by the activation of protein kinase A or C and decreased by the inhibition of these kinases [44].

Itraconazole, an antifungal agent (Figure 5), is known to cause drug-induced cholestasis (DIC). Yoshikado et al. have demonstrated that, in itraconazole-treated rats, biliary phospholipids, rather than bile salts, are drastically decreased and that the ABCB4-mediated efflux of [^14^C]PC from LLC-PK1 cells is reduced in the presence of itraconazole [45].

## 6. ABCB4-Mediated Efflux of Drugs

Initially, it was thought that ABCB4 is responsible for exporting various drugs due to its high homology with ABCB1 (Figure 2). However, Schinkel et al. have shown that ABCB4-expressing BRO human melanoma cells exhibit no resistance against a range of drugs including vincristine, colchicine, etoposide, daunorubicin, doxorubicin, actinomycin D, and gramicidin D [5]. Kino et al. have reported that the expression of ABCB4 in yeast confers resistance to aureobasidin A, an antifungal cyclic depsipeptide antibiotic, which is overcome by vinblastine, verapamil, and cyclosporine A [46]. The treatment of ovarian cancer cells with ABCB4 siRNA induces minor reduction in the paclitaxel resistance [47]. Smith et al. have reported that polarized monolayers of ABCB4-expressing LLC-PK1 cells show an increased directional transport of several ABCB1 substrates, such as digoxin, paclitaxel, daunorubicin, vinblastine, and ivermectin, and that the transport rate of these drugs, except for paclitaxel, is lower in ABCB4-expressing cells than in ABCB1-expressing cells [35]. Furthermore, ABCB4-dependent transport of digoxin is inhibited by ABCB1 reversal agents, cyclosporine A, valspodar, and verapamil [35]. Recently, we have demonstrated that the expression of ABCB1 or ABCB4 in HEK293 cells decreases the accumulation of rhodamine 123 and rhodamine 6G and that these reductions are more marked in ABCB1-expressing cells than in ABCB4-expressing cells [42]. The accumulation of BODIPY-verapamil in HEK293 cells is strikingly reduced by ABCB1 expression but is not altered by ABCB4 expression, indicating that BODIPY-verapamil is not a transport substrate of ABCB4 but an inhibitor of the ABCB4-mediated phospholipid efflux [42]. These findings suggest that ABCB4 cannot cause multidrug resistance due to the low rates of ABCB4-mediated export of drugs compared with ABCB1-mediated export. The nonphospholipid substrates may have lower affinities for ABCB4 than ABCB1 and/or compete with membrane PC for binding to ABCB4.

Furthermore, we have shown that the addition of taurocholate has no effect on the ABCB4-mediated efflux of rhodamine 123 and rhodamine 6G, which may be attributed to the sufficient solubility of these substrates in the aqueous medium [42].

## 7. ATP Hydrolysis by ABCB4

NBFs of all ABC transporters show extensive identity of amino acid sequence and conserved motifs, including the Walker A, Walker B, and signature motif (Figure 1) [48]. It has been assumed that the conformational changes at NBFs as a consequence of ATP binding and/or hydrolysis are transmitted to TMHs, leading to a high-affinity to low-affinity switch at the substrate-binding site. ATP binds at the interface of the two NBFs, which induces the formation of a closed dimer.

Smith et al. have confirmed the specific MgATP binding and the vanadate-dependent, *N*-ethylmaleimide-sensitive nucleotide trapping activity of ABCB4, using the radiolabeled photoaffinity ATP analog [*α*-^32^P]8-azido-ATP and insect Sf9 cell membranes overexpressing ABCB4 [35]. Vanadate replaces inorganic phosphate bound to ABCB4 and inhibits ATP-hydrolysis, which results in the formation of a complex between ADP and ABCB4 that cannot be dissolved by high MgATP concentrations. The nucleotide trapping by ABCB4 in the presence of vanadate is nearly abolished by EDTA, paclitaxel, vinblastine, verapamil, cyclosporine A, and valspodar, whereas the nucleotide trapping by ABCB1 is greatly stimulated by verapamil [35].

More recently, Ishigami et al. have constructed the chimera protein containing TMHs of ABCB1 and NBFs of ABCB4 and have analyzed the features of human ABCB4 NBFs [49]. Similar to ABCB1, the chimera protein confers the resistance against vinblastine and paclitaxel and mediates the calcein AM efflux but not the phospholipid efflux in the presence of taurocholate [49]. In the presence of vanadate, verapamil strongly enhances the ADP trapping of ABCB4 NBFs in the chimera protein as well as that of ABCB1 NBFs [49]. The ATPase activity of the purified chimera protein is stimulated by vinblastine and verapamil. However, the drug-stimulated ATPase activity of the purified chimera protein is much lower than that of purified ABCB1 [49].

## 8. Subcellular Localization of ABCB4

Fibrates are ligands for peroxisome proliferator-activated receptor *α*. In HepG2 cells, the treatment with bezafibrate has no effect on the levels of ABCB4 protein but induces the redistribution of ABCB4 into pseudocanaliculi between cells [36]. Ghonem et al. have recently reported that fenofibrate upregulates ABCB4 mRNA and protein expression in primary cultured human hepatocytes [37]. Receptor for activated C-kinase 1 (RACK1) is a 36 kDa cytosolic protein and can bind to various signaling molecules. ABCB4 protein, exogenously expressed in HeLa cells using the recombinant adenoviruses, is located dominantly on the plasma membrane and only a minor portion is observed intracellularly [50]. Downregulation of RACK1 expression by siRNA results in the localization of* ABCB4* in the cytosolic compartment, suggesting that RACK1 is involved in the trafficking of ABCB4 from the Golgi to the plasma membrane [50].

The mutation I541F, located in the first NBF of ABCB4, has been described in a homozygous patient with PFIC3 [19]. ABCB4 is localized at the pseudocanalicular membrane in HepG2 cells or at the apical surface in MDCK cells, whereas the I541F mutant is retained intracellularly [19]. After shifting cells to 27°C, the intracellular traffic of this mutant is restored [19]. In addition, cyclosporine A allows a significant amount of the I541F mutant protein to reach the pseudocanalicular membrane in HepG2 cells [51]. The S320F variant is linked with the development of cholestatic disorders including ICP, LPAC, DIC, and PFIC3, and the A953D mutation is found in heterozygosity with the S320F mutant [52]. The transient expression of the S320F or A953D mutant is low at the plasma membranes in HEK293 cells, but cyclosporine A improved the plasma membrane localization of both mutants [52]. Two mutations, G68H and D459H, have been identified in children with PFIC3 and result in the retention of ABCB4 in endoplasmic reticulum in MDCK cells [53].

Lipid rafts are small (10–200 nm) plasma membrane domains containing high levels of sphingolipids, mainly SM, and cholesterol, which are characterized physicochemically by tight packing and reduced fluidity leading to a liquid-ordered phase surrounded by the bulk liquid-disordered membranes [54–56]. Recently, we have investigated the relationships between the functions of ABCB4 and lipid rafts [42]. To isolate the lipid rafts, we have used Triton X-100 insolubility assay and OptiPrep gradient centrifugation method [57, 58]. In mouse canalicular membranes, Abcb4 is exclusively localized to the nonraft membranes [42]. Likewise, in ABCB4-expressing HEK293 cells, ABCB4 is predominantly distributed into the nonraft membranes [42]. ABCB4 expression leads to significant increases in the contents of PC, PE, and SM in nonraft membranes and to further enrichment of SM and cholesterol in raft membranes [42]. The ABCB4-stimulated efflux of PC, PE, and SM in the presence of taurocholate is completely abolished by BODIPY-verapamil, which partitions hardly into the raft membranes [42]. Collectively, these results have indicated that the taurocholate-stimulated phospholipid efflux is mediated exclusively by ABCB4 located in the nonraft membranes.

## 9. Molecular Model for ABCB4-Mediated Flopping and Efflux

ABCB1 and ABCB4 are 86% similar in terms of amino acid sequence (Figure 2). The crystal structures of ligand-bound mouse Abcb1a and ADP-bound sav1866, a bacterial homolog of ABCB1, have been published [59, 60]. The nucleotide-free but ligand-bound ABCB1 structure represents the inward-facing conformation, which is formed from two bundles of six TMHs and two separated NBFs [60]. The inward-facing structure has a large internal pocket open to both the cytosol and the inner leaflet and does not allow substrate access from the outer membrane leaflet or the extracellular space. Binding and/or hydrolysis of ATP have been thought to trigger a conformational switch opening the binding pocket to the outer leaflet and external aqueous environment and to reduce the affinity for the ligand [61]. After ATP hydrolysis and dissociation of the ligand, the TMHs reset to the inward-facing conformation.

**Figure 6:**
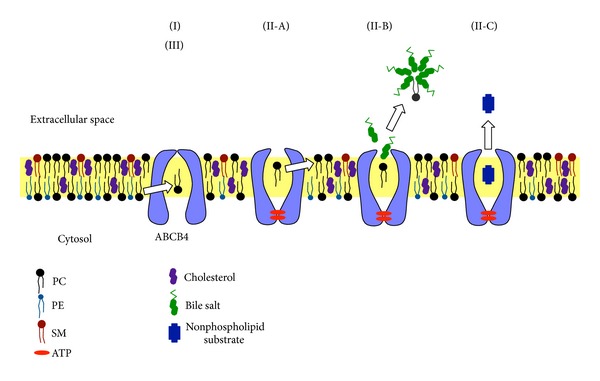
Model of molecular mechanism of ABCB4-mediated transport. (I) A substrate, mainly PC, enters the binding pocket of ABCB4 from the inner leaflet through the gaps between TMHs. (II) Binding and/or hydrolysis of ATP trigger a conformational change opening the binding pocket to the outer leaflet and the extracellular space. (II-A) In the absence of bile salts, a PC molecule laterally diffuses from the binding pocket of ABCB4 to the outer leaflet through the gaps between TMHs, which represents a floppase function of ABCB4. (II-B) In the presence of bile salts, a PC molecule is taken up from the binding pocket of ABCB4 by bile salt monomers, which represents an exporter function of ABCB4, and then a mixed bile salt/PC micelle is formed in the extracellular space. (II-C) A substrate with sufficient aqueous solubility directly diffuses from the binding pocket into the extracellular space regardless of the presence or absence of bile salts. (III) After dissociation of the substrate and ADP molecules, ABCB4 reset to the inward-facing state.

On the basis of the information inferred from the crystal structures and experimental evidence, we propose the following model for the molecular mechanism of the ABCB4-mediated flopping and efflux (Figure 6). (I) As a first step, a substrate, mainly PC, enters the binding pocket of ABCB4 from the inner leaflet of nonraft membrane through the gaps between TMHs. Following the conformational change induced by ATP binding/hydrolysis, (II-A) in the absence of bile salts, a phospholipid molecule laterally diffuses from the binding pocket of ABCB4 to the outer leaflet of the membrane through the gaps between TMHs; (II-B) in the presence of bile salts, a phospholipid molecule is picked up by bile salt monomers, and then a mixed bile salt/phospholipid micelle is formed in the extracellular space; and (II-C) a nonphospholipid substrate with sufficient aqueous solubility diffuses directly into the extracellular space regardless of the presence or absence of bile salts. Thus, we speculate that ABCB4 has a dual role as a floppase or as an exporter, depending on the presence of bile salts and on the aqueous solubility of the substrate.

ABCB4 has been predicted to be a floppase that translocates phospholipids from the inner leaflet to the outer leaflet of the canalicular membrane [32, 34, 62–65] or to be a transporter that moves phospholipids to be directly extracted by bile salts [33, 38]. However, the floppase activity of ABCB4 for long-chain PC has not been directly observed, although the ABCB4-mediated translocation of fluorescent-labeled short-chain PC analog (C_6_-NBD-PC) in yeast secretory vesicles [32, 33] and the release of C_6_-NBD-PC from ABCB4-expressing epithelial cells into the apical albumin-containing medium [34, 35] have been demonstrated previously. Meanwhile, Crawford et al. have observed abundant unilamellar vesicles in the bile canaliculi of* Abcb4* (+/+) mice but not* Abcb4* (−/−) mice and proposed that the biliary phospholipids are secreted as vesicles, which are formed by the ABCB4-mediated translocation of PC to the outer leaflet of the canalicular membrane and subsequently by the destabilization of the membrane by bile salts [63]. In contrast, Oude Elferink and Paulusuma have suggested that the biliary PC excretion takes place by the translocation followed by exposition of the PC molecule by ABCB4, which subsequently allows the extraction by bile salt micelles [4].

We consider that taurocholate monomers can access to the substrate-binding pocket of ABCB4 owing to their small size and take up phospholipid molecules directly from the binding pocket. The association of taurocholate monomers with a phospholipid molecule should reduce the activation energy required to move a phospholipid from the binding pocket of ABCB4 to the aqueous environment. It is also possible that taurocholate monomers directly interact with the amino acids lining on the binding pocket of ABCB4 and help the release of a phospholipid molecule because the hydrophilic bile salt, taurocholate, can induce the ABCB4-mediated secretion of phospholipids more efficiently than the hydrophobic bile salt, cholate.

## 10. Future Directions

In this review, we summarize current knowledge of the molecular properties of ABCB4 and its physiological relevance and discuss possible mechanism for the phospholipid efflux mediated by ABCB4. Clearly, further studies are required to elucidate the molecular mechanism. At present, it is largely unknown how taurocholate molecules support the phospholipid secretion mediated by ABCB4 or why ABCB4 is highly specific for PC, unlike ABCB1. Nonetheless, the lipid transport mechanism of ABCB4 may be less complex than the mechanisms of other ABC lipid transporters, such as ABCA1, ABCA3, ABCA12, ABCG1, and ABCG5/ABCG8. Accordingly, investigations into the molecular function of ABCB4 will also help to clarify the mechanisms of other ABC lipid transporters.

## Abbreviations

ABC: ATP-binding cassette
DIC: Drug-induced cholestasis
ICP: Intrahepatic cholestasis of pregnancy
LPAC: Low-phospholipid-associated cholelithiasis
MDR3: Multidrug resistance 3
NBF: Nucleotide-binding fold
PC: Phosphatidylcholine
PE: Phosphatidylethanolamine
PFIC3: Progressive familial intrahepatic cholestasis type 3
RACK1: Receptor for activated C-kinase 1
SM: Sphingomyelin
TMH: Transmembrane helix.
